# Dynamic changes in purine catabolism in patients with acute coronary syndrome that underwent percutaneous coronary intervention

**DOI:** 10.22088/cjim.10.1.86

**Published:** 2019

**Authors:** Olga Visternichan, Seyed Farzad Jalali, Dana Taizhanova, Larissa Muravlyova, Gaukhar Igimbayeva

**Affiliations:** 1 Karaganda State Medical University, Karaganda, Kazakhstan; 2 Health Research Institute, Babol University of Medical Sciences, Babol, Iran

**Keywords:** Coronary heart disease, Acute coronary syndrome (ACS), Purine catabolites, Uric acid, Percutaneous coronary intervention (PCI).

## Abstract

**Background:**

Cardiovascular diseases are global problems. They are causes of death in about 43% of people worldwide and may become the most widespread reason of death by 2020. The prognosis is directly dependent to immediate diagnosis and on time treatment. Introduction of new biochemical markers as the early diagnosis of complications after coronary revascularization is very important in this period. Herein, we assayed the changes of purine catabolites in patients with acute coronary syndrome (ACS) before and after percutaneous coronary intervention (PCI) in comparison with control group.

**Methods:**

Thirty five ACS patients (20 males and 15 females) were included (57±17 years old) in the study. The determination of intermediates of purine catabolism as guanine, hypoxanthine (GCS), adenine, xanthine (Kc) and uric acid (MK) were assayed before and 3 days after PCI. Conditionally, 35 healthy-matched persons were included in the control group. Purine catabolites were determined in plasma through the method of Oreshnikov E.V (2008).

**Results:**

In ACS patients, prior to PCI, there was a tendency to increase the concentration of guanine (P=0.001), hypoxanthine (P=0.002) adenine (P=0.0003), xanthine (P=0.000003) and uric acid (P=-0.000001) relative to the upper limits of normal ranges. And on the third day after PCI, there was the second tendency to increase the levels of guanine (P=0.000001), hypoxanthine (P=0.000001) adenine (P=0.0000001), xanthine (P=0.000001) and uric acid (P=0.0000001) relative to upper limits of normal ranges.

**Conclusion:**

Increment of plasma purine catabolites can be a marker of inflammation and instability of coronary artery plaques and may be used as a restenosis marker in patients with history of PCI.

Cardiovascular diseases are not only big medical problems but also have social consequences. Ischemic heart diseases (IHD) are cause of death in about 43%of people worldwide, but according to predictions, it might become the most widespread cause of death by 2020 (1). Today in Kazakhstan, cardiovascular diseases are the cause of death in one-third of people, and as well in the previous decade, the morbidity of IHD has increased 5-7 times* (*2*). *In the development and progression of coronary heart diseases, some factors have great roles such as increased population age, unusual habits, nutrition and genetic factors (3). The prognosis is directly related to immediate diagnosis, prevention of complications and urgent myocardial revascularization (4). 

There are some risks to develop complications in spite of the improvement in modern interventional cardiology and cardiac surgery. More often these complications resulted in activation and progression of inflammatory processes in atherosclerotic or PCI zones (5). 

Evaluation of the role of extracellular purines in the development of various pathological conditions is a new tendency in modern biochemistry. Adenine and guanine are the basic purine bases. Xanthine and hypoxanthine are their catabolites, and uric acid is the final product of purine catabolism. Purines have influence on the cells with activation of specific receptors on the cell membrane. Such receptors are in majority number in blood vessels, heart and other organs in the body (6). In particular, the different types of Р2Y-receptors are revealed in progression of heart failure. It determines their role in myocardial function (7). On the other hand, hyperuricemia initiates aseptic inflammation (8). Marked hyperuricemia unfavorably affects the hemostatic system and vascular wall (9). 

The aim of this research was to assess the dynamic changes of purine catabolites in blood plasma of patients with acute coronary syndrome before and after percutaneous coronary intervention. 

## Methods

35 ACS patients (17 females, 18 males) were included (57±17 years old)in the study. These patients with acute coronary syndrome were admitted to the Emergency Cardiac Department of Karaganda City Hospital. Diagnosis of ACS was done by expert cardiologists via history taking, physical examination, electrocardiography, echocardiography, cardiac biomarkers like troponin I and T, and coronary angiography. Levels of plasma purine concentrations were assayed before and 3 days after PCI. The patients with diabetes mellitus, chronic obstructive pulmonary disease, gout, chronic rheumatic heart disease with formation of valvular defects, cancer, as well as patients with renal dysfunction were excluded. 

Conditionally 35 healthy matched persons (20 females, 15 males), (55±5 years old) were included in the control group. Determination of intermediates of purine catabolism as guanine, hypoxanthine (GCS), adenine, xanthine (Kc) and uric acid (MK) were assayed in plasma of all cases and control group. Venous blood was used as a research material, and the metabolites of purine were determined by the method of Oreshnikov E.V. et al. (2008) (5). Concentration of purine catabolites was expressed in terms of extinction (unit of ext.), and the concentration of uric acid in μmol / l. Statistical analysis of the data was performed using the software package STATISTICA Version 8.0 based on the computational methods recommended for biology and medicine. The analysis of the obtained data included the calculation of average arithmetic variation series (M) and its error (m). Reliability of observed differences was determined by paired t-test method using the t-coefficient of Student.

## Results

We evaluated the purine catabolites in patients with acute coronary syndrome before and on the 3rd day after PCI in comparison with the control group, and the statistical significant differences were revealed (table 1).

**Table 1:** Mean values (standard deviation) of purine metabolites in patients with ACS before and on the 3rd day after PCI compared with control group

	**Mean(standard deviation)** **Before PCI**	**Mean(standard deviation)** **3 days after PCI**	**Mean(standard deviation)** **control group**
Guanine	0.47 (0.53)*	1.33 (0.96)*	0.14 (0.04)*
Hypoxanthine	0.42 (0.54)*	1.24 (0.99)*	0.12 (0.04)*
Adenine	0.28 (0.26)*	0.84 (0.77)*	0.1 (0.04)*
Xanthine	0.26 (0.12)*	0.45 (0.29)*	0.14 (0.06)*
Uric acid	0.28 (0.09)*	0.36 (0.14)*	0.16 (0.05)*

* Statistical significant differences via a student t-test for independent samples (р<0.05)

**Figure 1:**
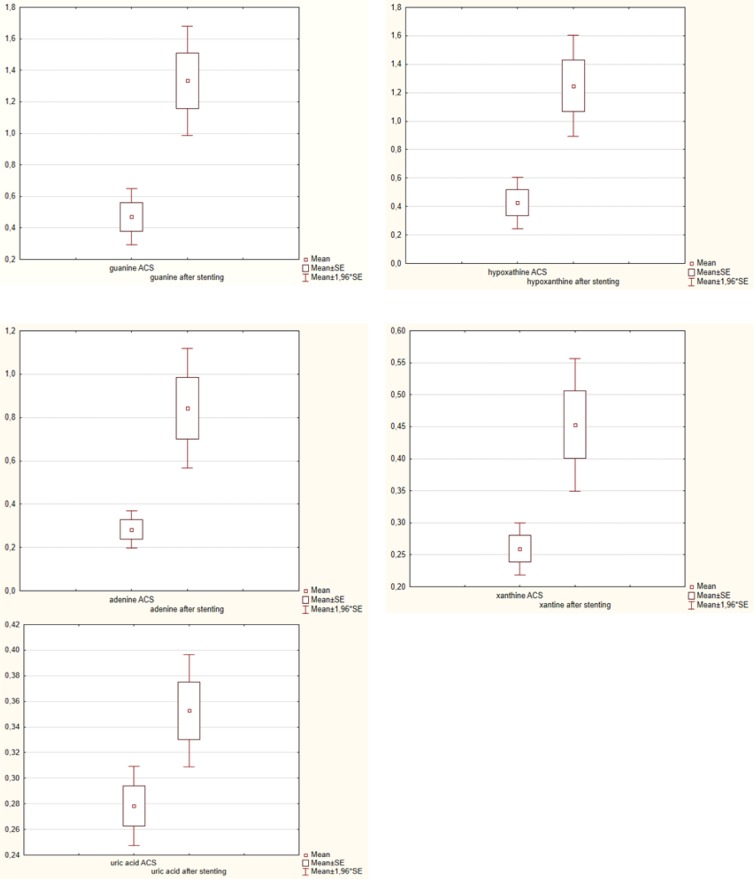
Dynamic changes in plasma purine catabolites before and after PCI (stenting) in ACS patients

An apparent increment was observed in plasma concentrations of guanine, hypoxanthine, adenine, xanthine and uric acid in patients compared the with control group. In plasma samples taken from patients with acute coronary syndrome prior to PCI, there was a tendency to increase concentration of guanine (p-0.001), hypoxanthine (p-0.002) adenine (p-0.0003), xanthine (p-0.000003) and uric acid (p-0.000001) relative to the physiologic upper limits. In plasma samples taken from the same patients on the third day after PCI, there was also second tendency to increase the content of guanine (p-0.000001), hypoxanthine (p-0.000001) adenine (p-0.0000001), xanthine (p-0.000001) and uric acid (p-0.0000001) relative to physiologic upper limits too. In comparison, there was a tendency to increase their concentration on the 3^d^ day after PCI. The results are shown in figure 1.

## Discussion

 Marked tendency to the elevation of purine catabolites in plasma of patients with acute coronary syndrome may be the result of various mechanisms. First of all, this can be due to entry into blood as result of cell damage during myocardial ischemia. Another reason may be a violation of the reverse transport of purine nucleotides and its intermediates into cells, which is caused by a violation of their capture by specific receptors on cell membranes. In addition, the development of acute coronary syndrome is preceded by processes leading to atherosclerotic plaque to become unstable. A similar plaque related to infarction, more often has non-dramatic character. That is why it does not lead to significant disruption of the coronary blood flow (2). Unstable plaque is abundantly infiltrated by inflammatory cells, which contribute to the damage of fibrous cap. As a result of atherosclerotic plaque rupture or of its erosion, platelet aggregation inducers (collagen and other substances) and tissue factor inducers are released; and these lead to the start of thrombogenesis process. In the process of inflammation, a large number of purine metabolites are released into the extracellular space by neutrophils, endothelial cells and activated macrophages. It explains an increase in the concentration of purine catabolites in plasma. ADP, which is released by platelets, also has the great contribution into the elevation of the level of extracellular purines after dephosphorization (10). Significant increase in the concentration of purine catabolites after PCI may be due to the development of aseptic inflammation in the stented area, which is also accompanied by the migration of inflammatory factors to this part of coronary artery (8).

Adverse effects of hyperuricemia on hemostatic system and vascular wall were established (11). Larina V.N. et al. in their scientific investigation in 2016, pointed to the role of chronic tissue hypoxia in cardiovascular diseases and in the development of endothelial dysfunction, which in turn leads to an increment in the activity of xanthine oxidase enzyme and the accumulation of uric acid as the final product of purine metabolism, in the plasma (6). 

 In this research, we evaluated complete spectrum of intermediate purine metabolites in the blood plasma of patients with acute coronary syndrome before and after PCI.

The results of our investigation showed that in patients with acute coronary syndrome before and on the 3rd day after PCI, the elevation of all purine catabolites in blood plasma were present, with marked elevation in metabolites of initial stage of catabolism of purines: adenine, guanine and hypoxanthine. It may be the result of low activity of the specific ferment – xanthine oxidase. 

Some researchers like Don E Farthing et al. describe the significant contribution of necrosis and lysis of cardiomyocytes in the elevation of extracellular concentration of intermediates of purine metabolism. This pathological process is associated with the next mechanisms (11). For normal function of cardiac muscle, it is necessary to use much amount of ATP produced by intracellular mitochondria in cardiomyocytes. About 80% of all ATP in myocardium is produced by such organelles using aerobic oxidative phosphorylation in electron transport chain. The normal function of this process is associated with sufficient number of normally functioning cardiomyocytes under sufficient oxygenation. Therefore, the development of acute ischemia as the result of insufficiency of coronary arteries, lead to severe metabolic disturbances in the affected myocardium (12).

At the onset of cardiac ischemia, the high energy phosphates (creatine phosphate and ATP) are rapidly depleted and heart tissues would lose 65% of their ATP contents within 15 minutes of complete ischemia (13). Such ischemic events in turn mobilize ATP breakdown cascade (14) that leads to cellular accumulation of ATP catabolic by-products, including adenosine diphosphate (ADP), adenosine monophosphate (AMP), and activates normally dormant enzymes, such as 5′-nucleotidase, adenosine deaminase, purine nucleoside phosphorylase, and xanthine oxidase, which sequentially catabolize AMP into adenosine, inosine, hypoxanthine, xanthine, and uric acid. Upon reperfusion of the heart with oxygenated blood or perfusate, xanthine oxidase and xanthine dehydrogenase convert hypoxanthine to xanthine and uric acid (14).

Furthermore it is known that inosine and hypoxanthine are the small molecules with molecular mass about 268 Dalton for inosine and 136 Dalton for hypoxanthine. This fact facilitates their rapid passive diffusion to the plasma from damaged cardiomyocytes (14). Thus, the described mechanisms explain the statistically significant elevation of extracellular concentration of purine catabolites in patients with acute coronary syndrome.

The next important moment determined during our investigation, is the tendency to a significant increase in the concentration of all purine catabolites on the 3rd day after PCI. What is the reason of such changes? 

Ghaemi-Oskouie, et al. (15), connected with estimation of the role of uric acid as an endogenous danger signal in immunity and inflammation, determined that uric acid has been identified as an endogenous adjuvant that drives immune responses in the absence of microbial stimulation. Because uric acid is an ubiquitous metabolite that is produced in high quantities upon cellular injury, the ramifications of its effects may be considered. Uric acid crystals also have been shown to trigger interleukin-1β–mediated inflammation via activation of NOD-like receptor protein (NLRP) 3 inflammasome, a multimolecular complex whose activation appears to be central to many pathological inflammatory conditions (16).

In other studies (10, 17), the authors proved that hyperuricemia may act as the initiator of aseptic inflammatory reactions in various tissues including the myocardium and endocardium. As a result, based on many researches, considering the possible damage to intima of coronary artery during PCI, the development of aseptic inflammation in the vascular wall leads to endothelial dysfunction and an increase of extracellular concentration of purines, including uric acid. In its turn, hyperuricemia will also contribute to stimulation of inflammatory process, which can provoke progression of the atherosclerotic process in the stented area, the formation of neointima and in-stent restenosis as final result.

In conclusion, the analysis of obtained data shows the need to further studies about extracellular purine involvement in the development and progression of coronary heart disease. In the case of acute coronary syndrome, purine catabolites act as the so-called "anxiety molecules". Elevation of their concentration signals cellular damage of the vascular endothelium under the situation of severe hypoxia. Significant increase in their concentration after PCI may be the marker of an inflammatory reaction in the endothelium of the coronary vessels due to the damage of plaque during PCI. It may provoke further progression of the inflammatory processes after coronary revascularization and contribute to the development of restenosis of vessel.
